# Lab-on-chip microscope platform for electro-manipulation of a dense microtubules network

**DOI:** 10.1038/s41598-022-06255-y

**Published:** 2022-02-14

**Authors:** Daniel Havelka, Ilia Zhernov, Michal Teplan, Zdeněk Lánský, Djamel Eddine Chafai, Michal Cifra

**Affiliations:** 1 Institute of Photonics and Electronics of the Czech Academy of Sciences, Prague, Czechia; 2 BIOCEV, Institute of Biotechnology of the Czech Academy of Sciences, Prague, Czechia; 3 Institute of Measurement Science of the Slovak Academy of Sciences, Bratislava, Slovakia; 4 Institute of Physiology of the Czech Academy of Sciences, Prague, Czechia

**Keywords:** Nanoscale biophysics, Electrical and electronic engineering, Biological physics

## Abstract

Pulsed electric field (PEF) technology is promising for the manipulation of biomolecular components and has potential applications in biomedicine and bionanotechnology. Microtubules, nanoscopic tubular structures self-assembled from protein tubulin, serve as important components in basic cellular processes as well as in engineered biomolecular nanosystems. Recent studies in cell-based models have demonstrated that PEF affects the cytoskeleton, including microtubules. However, the direct effects of PEF on microtubules are not clear. In this work, we developed a lab-on-a-chip platform integrated with a total internal reflection fluorescence microscope system to elucidate the PEF effects on a microtubules network mimicking the cell-like density of microtubules. The designed platform enables the delivery of short (microsecond-scale), high-field-strength ($\le$ 25 kV/cm) electric pulses far from the electrode/electrolyte interface. We showed that microsecond PEF is capable of overcoming the non-covalent microtubule bonding force to the substrate and translocating the microtubules. This microsecond PEF effect combined with macromolecular crowding led to aggregation of microtubules. Our results expand the toolbox of bioelectronics technologies and electromagnetic tools for the manipulation of biomolecular nanoscopic systems and contribute to the understanding of microsecond PEF effects on a microtubule cytoskeleton.

## Introduction

Technology for advancing the understanding of the interactions between an electromagnetic field and biomatter is important for the development of novel approaches for the manipulation of molecular function as well as elucidating the bioeffects of electromagnetic fields, which could lead to new biomedical therapy protocols. The most desirable methods should enable real-time in situ imaging of the electromagnetic field effects on molecular structures. However, that requires the integration of advanced microscopy techniques with properly engineered chip technology that enables the delivery of a precisely localized electromagnetic field. To address these challenges, in this paper, we describe the development of an advanced optical-microscopy-integrated platform that combines chips capable of delivering a pulsed electric field (PEF) to microtubules (MTs) during real-time imaging.

MTs are polymers that are present in the cytoskeleton of cells. They are formed by the self-assembly of tubulin protein into hollow tubes with a 25-nm outer diameter and typical lengths from a few hundred nanometers to several tens of micrometers^1^. MTs are crucial for cell morphogenesis, cell motility, cell division, and intracellular transport. MT-related malfunctions are associated with a spectrum of neurological^2,3^ and psychiatric^3,4^ diseases, and MTs are also an important therapeutical target in cancer treatment^5^. MT-associated proteins also represent a natural nanoscopic toolbox for functions such as self-assembly^1,6^, force generation^7,8^, and transport on the nanoscale^9^. MTs have unusual electrical properties^10^. *In silico* experiments have shown that their building blocks, tubulins, have a much higher net structural electric charge and dipole than most other proteins^11^, which suggests that MTs may be susceptible to an electric field (EF), possibly affecting their structure and function. Here we focus on PEF, which has been increasingly applied in biomedicine^12,13^, the food industry, and biotechnology^14,15^. It has been demonstrated that PEF affects the MT cytoskeleton^16^. In PEF research, the pulse duration is an important parameter. This is because it determines the pulse spectral energy content, which affects how the biological matter responds to PEF. In the range of pulse lengths > 100 $\upmu$s, the application of PEFs to cells has been shown to have varying effects on MTs: fragmentation^17^ or denser packing^18^ of MTs, depending on the conditions. In the nanosecond pulse length range, PEFs have been shown to disrupt the MT network, either immediately^19^ or, if the complexity of the intracellular environment obscures the direct effect (if any) of PEF on the MTs from the indirect downstream effect induced by the accompanying processes, with a delay^20^. In addition, we have recently established an approach to remodel MTs in cells without completely disassembling them^21^.

**Figure 1:**
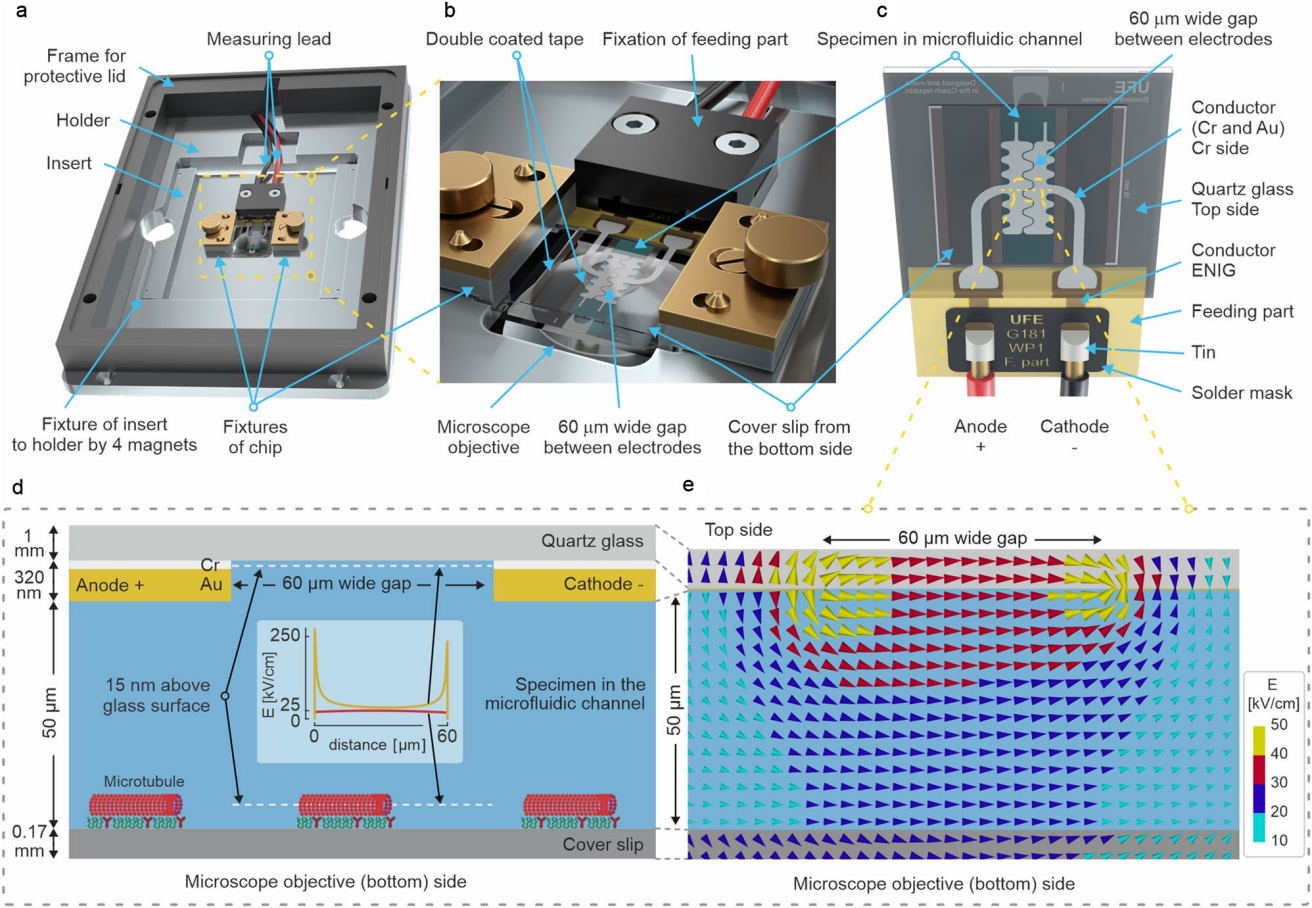
Chip platform design and electric field simulations. **(a)** A model of the chip platform with a frame for the protective lid. **(b)** Detail of the fixed chip with a microfluidic channel in electrical contact with the fixed electrical feeder on the insert under supervision of the microscope objective from the bottom. **(c)** Detail of the chip with a microfluidic channel and electric feeder. **(d)** A description of the perpendicular cross-section through the microfluidic channel in the area of the 60 $\upmu$m gap between two electrodes (anode and cathode). The graph in the inset depicts a comparison of electric field strength calculated on the 60 $\upmu$m long white dashed lines localized 15 nm from both the top and bottom of the microfluidic channel. **(e)** A calculated time snapshot of the 2D electric field strength distribution (arrows indicate electric field vector) in the cross-section described in **(c)** excited by a 300 V pulse.

However, the effects of PEF in the range of a few microseconds ($\upmu$s-PEF) on MTs have not been explored. Furthermore, there is no data on the direct effects of PEF on MTs: the reported effects on MTs in cells might be indirect downstream effects from membrane permeabilization^22^, action on membrane channels^23^, or changes in intracellular ion concentration^24^. Although several PEF chip-microscope integration technologies have been developed in recent years^25–27^, none of them demonstrate the capability of $\upmu$s-PEF delivery to out-of-the-cells MT networks in a microfluidic environment. To fill in these technology and knowledge gaps, we present here the design, fabrication, and verification of a new chip assembly, embedded to a total internal reflection fluorescence (TIRF) microscope, that enables the delivery of $\upmu$s-PEF. Using this new experimental platform, we then demonstrate in vitro that $\upmu$s-PEF can influence multivalent interactions between MTs and the substrate.

## Results and discussion

**Figure 2:**
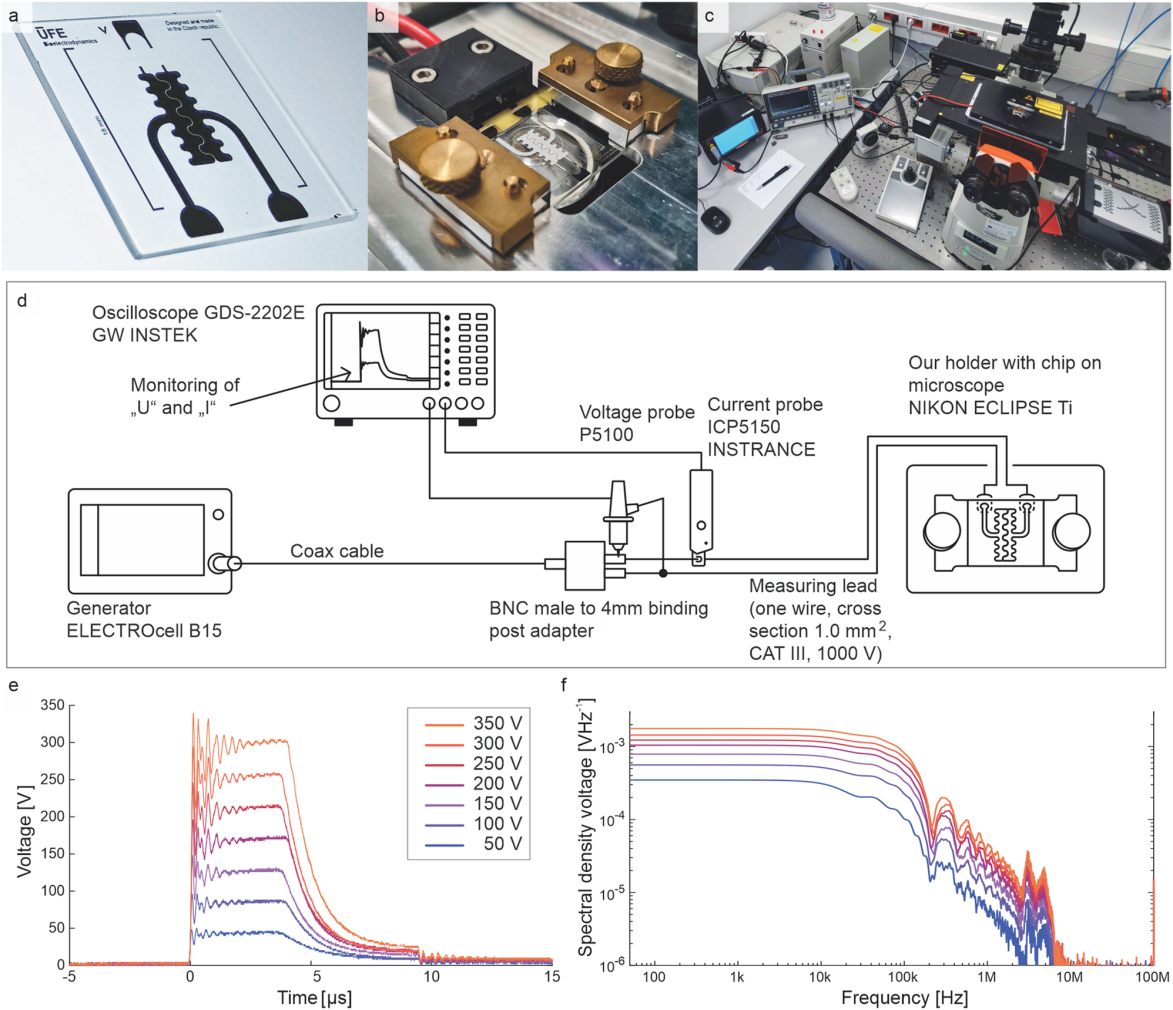
Fabricated chip platform and experimental setup. **(a)** Fabricated chip. **(b)** Fabricated chip platform mounted on a Nikon Eclipse Ti TIRF microscope stage. **(c)** A photograph of the experimental pulse electric field setup. **(d)** A scheme of the experimental pulse electric field setup. **(e)** Typical pulse shapes of used pulses, legend shows the voltages set on the electric pulse generator. **(f)** Voltage spectral density recalculated from recorded pulses.

### Chip platform design and simulation

In this section, we describe the development of a new $\upmu$s-PEF-chip microscopy platform that can be reproducibly fixed to a Nikon Eclipse Ti TIRF microscope stage and enables easy manipulation of the chip and delivery of $\upmu$s-PEF to out-of-the-cell MT networks. The standard flow chamber for the TIRF microscope MT essay is composed of two parallel thin cover glasses stuck together by wax strips, creating a microfluidic channel with a typical height of approximately 100 $\upmu$m. Both the lower (the one closer to the objective) and upper glass are covered with MTs. We made two changes to this standard design. The first modification was to replace the wax strips with double coated tape with a thickness of 50 $\upmu$m to decrease the channel height. The second modification was to substitute the cover glass farther from the objective with a thicker glass which contained two thin-film gold electrodes, which allowed the delivery of a high electric field strength to the microtubule mesh at a low voltage (<1 kV) pulse via a 60 $\upmu$m wide gap between the electrodes. To implement these two modifications and achieve the technological goals, such as easy mechanical manipulation of the setup, reproducible electrical contact, and high field strength close to the microscope objective, our chip platform was composed of three main parts: a holder, an insert, and a chip with a microfluidic channel (Fig. 1). The largest part was a holder that was compatible with the microscope table and had a frame for the protective lid and the insert. The holder was made of aluminum, fixed to the microscope by screws, and was universally designed for holding different types of inserts. Mechanically, the most complex designed part was the insert, which enabled a reproducible electrical connection between the electrical feeder and the chip with a microfluidic channel and did not interfere with the microscope objective. The insert was made of aluminum and was fixed to the holder with four magnets. The electrical feeder was connected with two single measuring leads to a 15$\times$24$\times$0.5 mm$^3$ big single sided printed circuit board made from Isola E-Cu substrate and fixed to the insert with a black Teflon holder, which also protected the open electrical path. Two easily removable fixtures of the chip provided enough pressure to the electrical connection between the electrical feeder and chip with a microfluidic channel. The chip consisted of two thin-film gold serpentine electrodes deposited on the 25.4 × 25.4 × 1 mm$^3$ big quartz glass substrate (Figs. 1c, 2a), a double coated-tape to ensure the 50 $\upmu$m height of the microfluidic channel, and a 170 $\upmu$m thick cover slip on the the lower portion of the chip facing the microscope objective. The 60 $\upmu$m gap between the electrodes is wide enough to potentially accommodate even long MTs. We designed a gap with a regular serpentine shape for three reasons: (i) it enables a longer channel on a limited footprint than a straight channel, (ii) all angles of the electric field vector to the MT axis are ensured by this geometry, and (iii) no sharp edges are present on the horizontal footprint.

The drawback of placement of the gold electrodes on the glass substrate farther from the objective and attaching MTs on the cover glass close to the objective is that it is difficult to determine the exact location of the gap between the electrodes during MT observation with the TIRF microscope. This problem was solved by following the laser beam going through the gap between the gold electrodes. The flow chamber design that we implemented also provides a few benefits: (i) It is easier to fabricate a gold pattern on the thicker glass substrate instead of on a cover slip with a 170 $\upmu$m thickness. (ii) There is no change in the standard preparation procedure of cover glass with MTs. (iii) It is not possible to separate two cover glasses connected together with 4 strips of double coated tape without breaking them. Therefore, our chips can be recycled at least 10 times using a proper cleaning procedure. (iV) Near a gold pattern with a thickness of 320 nm, there is an inhomogeneous electric field strength distribution across the 60 $\upmu$m wide gap; therefore, it is beneficial to put the MTs farther from the gap.

**Figure 3:**
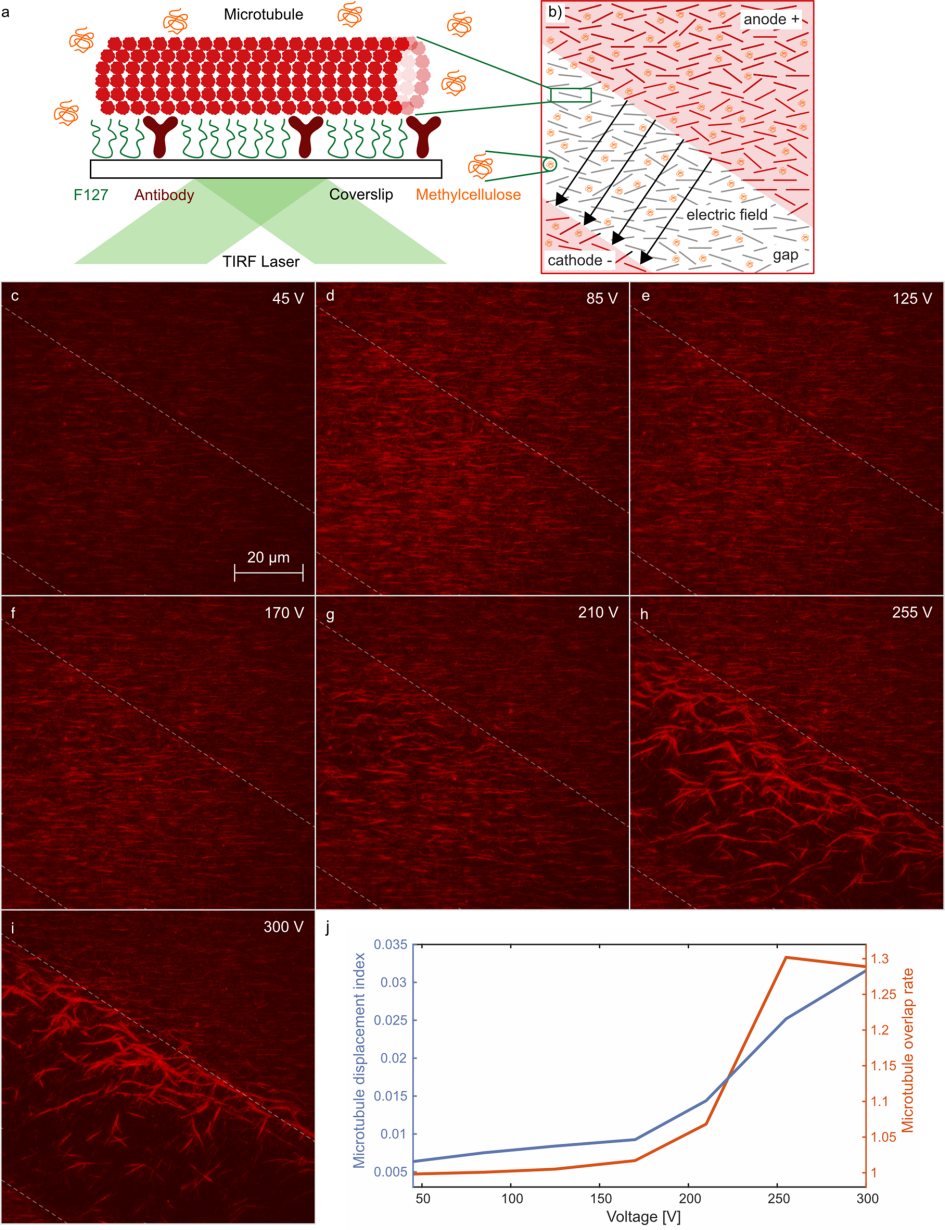
Pulse voltage dependence of microtubule displacement. A series of 5 $\upmu$s, N $=$ 100 pulses, with 10 Hz firing frequency were applied at each voltage. **(a)** MTs are bound to the coverslip surface via antibodies. **(b)** Scheme of the microscope field of view covering the gap between the electrodes, **(c)**–**(i)** snapshots from TIRF microscope imaging show MTs (red) after $\upmu$s-PEF treatment. Dashed lines represent approximate position of projection of the borders of the electrodes to the image plane. **(j)** *Microtubule displacement index* and *microtubule overlap rate*. The data in this figure was collected sequentially on the same chip, sample, and field of view, starting with 45 V, then 85 V, 125 V, 170 V, 210 V, 255 V, and 300 V; thus, the observed effects were cumulative.

The chip connected with double coated tape to the cover glass and a 4.4 $\upmu$L buffer inside the microfluidic channel were used for simulation of the electric field distribution with a low-frequency time domain solver in CST MWS (Dassault Systèmes^®^, Computer Simulation Technology Microwave Studio^®^). The buffer filled the whole microfluidic channel and was modeled as $\sigma$ = 0.1188 S/m (measured by Conductivity meter SevenCompact S230 with Cond. probe InLab 751-4mm, Mettler Toledo) and $\varepsilon _r$ = 80 (estimated from pure water). In computational modeling (CST MWS), we tested the effect of $\varepsilon _r$ change on the electric field strength in the gap: we found that the variation of $\varepsilon _r$ from 40 to 800 changed the electric field strength by less than 0.5% difference (in the middle of the gap) in comparison to the scenario with $\varepsilon _r$ = 80. We were interested in how the electric field distribution looks inside the channel in the perpendicular cross-section in the middle of the gold serpentine electrodes after excitation with a pulse with an amplitude of 300 V. The expected result of the 2D distribution of the electric field strength in the cross-section shows a lower electric field intensity on the interface of the cover glass and buffer but the distribution of the electric field was much more homogeneous in comparison with the surroundings of a 60 $\upmu$m gap (see Fig. 1e and inset in Fig. 1d). Based on this computational simulation, for the 300 V pulse, the electric field strength at the MT plane was estimated to be 25 kV/cm. We expect that the field strength scales down linearly with the voltage: for example, pulses with amplitudes of 255, 210, 170 and 45 V correspond to electric field strength of 21.25, 17.5, 14.2, and 3.75 kV/cm, respectively. See the whole experimental setup in Fig. 2. The chip platform was embedded on a Nikon Eclipse Ti TIRF microscope stage and connected to the electric pulse circuitry. Then, the whole setup was assembled according to the proposed scheme: A high voltage pulse generator (ELECTROcell B15, Leroy Biotech) that delivers 5 $\upmu$s pulses through cables to the chip was used. The voltage and current traces were measured using a voltage probe (P5100) and a current probe (ICP5150, Instrance), respectively, connected to an oscilloscope (GDS-2202E, GW Instek). The measured and processed voltage data are plotted in Fig. 2e,f. For the settings of 50 V, 100 V, 150 V, 200 V, 250 V, 300 V, and 350 V in the pulse generator, voltages of 45 V, 85 V, 125 V, 170 V, 210 V, 255 V, and 300 V, respectively, were measured. Therefore, these measured voltage values were used for labeling the data in the remaining text.

### Development of measures for quantification of $\upmu$s-PEF effects on microtubules

In the experimental results described below, we observed the displacement and overlap of MTs as the effect of $\upmu$s-PEF, see next section. To quantify the impact of $\upmu$s-PEF on MT displacement, several approaches might be used. We first tried classical image processing together with segmentation of the MT objects. However, this approach was difficult to automate, as the background was inhomogeneous and quite noisy. In particular, the borders between the MT objects and background were not sharp enough to reliably recognize single MTs. Moreover, with such a number of MT objects moving in not quite a unidirectional and synchronous way, together with their mutual overlapping, the tracking procedure necessary for following the motion of single MTs is highly nontrivial, or even impossible under the given conditions. Because of these reasons, an alternative approach had to be developed.

**Figure 4:**
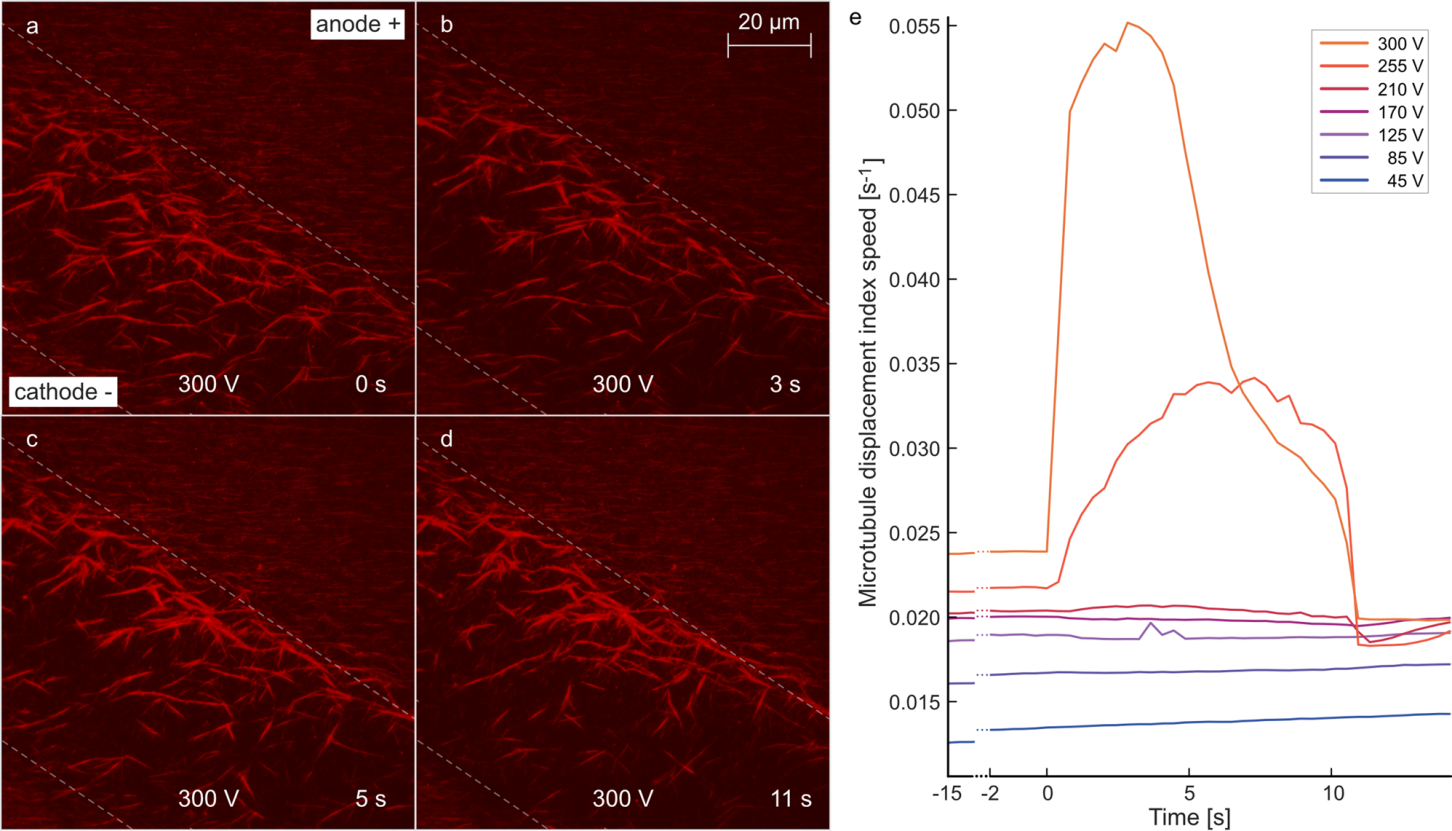
Time dependence of MT displacement by a sequence of electric pulses (5 $\upmu$s, N=100 pulses, 10 Hz firing frequency). **(a)**–**(d)** snapshots of fluorescently (red) labeled MTs during the firing of electric pulses at 300 V, **(e)** *Microtubule displacement index speed* depicting the displacement speed for each pulse voltage setting .

The goal was to statistically evaluate the overall movement of MTs without the necessity to recognize the individual objects. For this purpose, there was a need for a simple measure capable of quantifying the visible changes within each frame with respect to the previous one. Thus, the measure named *microtubule displacement index* (MDI) was constructed in the following way. It had to be able to detect all the places where some mass (represented by pixels of higher intensity recorded by TIRF microscopy) disappeared plus those places where it appeared. To achieve this, subtraction of the intensity matrices belonging to two time instants was realized. Then, an average from their absolute values within the region of interest (ROI) was computed:

1 $$\begin{aligned} MDI = \frac{1}{|ROI|} \sum _{[i\,j]\in {ROI}}{|I_{ij}(t_2)-I_{ij}(t_1)|} \end{aligned}$$

in which [i j] are matrix indices of intensity I. If we insert for $t_1$ and $t_2$ the times prior and after $\upmu$s-PEF application, the formula represents the overall MT displacement during the whole experimental run for a particular voltage (Fig. 3j). Explanatory illustrations can be found in Fig. S1.2. However, the same measure can be applied in a frame-to-frame manner (with successive $t_1,t_2$), providing insight into the dynamics of the displacement process (Fig. 4e). In such a case, the quantity represents actual time change, and after recalculation onto the second time scale we may term it *microtubule displacement index speed* [s$^{-1}$].

The proposed procedure is influenced by certain automatic adjustments of TIRF microscope camera, which resulted in brightness changes during the course of the recorded videos. Thus, the following correction had to be applied: Within each frame, a background area was secured, where MTs remained unaffected by $\upmu$s-PEF (Fig. 3b upper-right corner of the frame). The raw data in ROI (covering the rest of the frame) were corrected by division with the averaged background intensity on a frame-to-frame basis.

Next, we were interested in quantifying MTs’ overlap. In order to develop a new measure to provide insight into the formation of the overlaps, we followed a similar philosophy as during the creation of the previous measure. The idea is based on the counting of all pixels belonging to MTs in the ROI. The ratio of this quantity in time instant $t_1$ prior to the $\upmu$s-PEF application to the one obtained in time instant $t_2$ after the $\upmu$s-PEF application

2 $$\begin{aligned} MOR = \frac{no.\,\, of\, all\, MT\, pixels\, (t_1)> th}{no.\,\, of\, all\, MT\, pixels\, (t_2) > th} \end{aligned}$$

would (in case of exceeding a value of one) reflect formation of the overlaps. We termed the measure *microtubule overlap rate* (MOR) and its application is presented in Fig. 3j. For the need of identification of MT points, a transition from the gray scale to binary images was undertaken. The threshold *th* was determined by modification of the standard Otsu method. To illustrate the creation of MOR more clearly, a shift from lower towards higher light intensity values in the case of higher $\upmu$s-PEF voltages is presented in histograms (Figs. S1.3, S1.4).

### $\upmu$s-PEF effects on microtubules visualized by on-chip TIRF microscope platform

In this section we explore the direct effects of $\upmu$s-PEF on MTs. For the experiment, we attached fluorescently labeled MTs to the coverslip (Fig. 1d) through tubulin antibodies (Fig. 3a). We tuned the protocol and concentration so that a dense MT coverage was achieved, similar to the MT density in animal cells (several units to several tens of MTs /$\upmu$m$^2$^28,29^), to clearly demonstrate the electric field effect on a larger population of MTs. The orientation of the gap between the electrodes within the field of view is shown in the scheme in Fig. 3b. Before the application of $\upmu$s-PEF, the MTs were partially oriented along the direction of the flow (Fig. 3b) of the sample when it was injected into the microfluidic channel. For Figs. 3 and 4, the data was collected sequentially on the same chip, sample, and field of view, starting with 45 V, then 85 V, 125 V, 170 V (video S2), 210 V (video S3), 255 V (video S4), and 300 V (video S5); thus, the observed effects were cumulative. At each voltage, a sequence of N $=$ 100, 5 $\upmu$s pulses, fired at 10 Hz frequency, was applied. The rationale for the selection of these parameters was the following: the biological and biomolecular effects of $\upmu$s pulse-length range of PEF have been poorly explored so far^30,31^. Furthermore, a 5 $\upmu$s duration of the pulse was the shortest available with the given generator, and the combination of 10 Hz frequency with 100 pulses rendered a 10 second pulsing treatment period, which was long enough to obtain several tens of microscopy images (required for further analysis) at the available imaging frame rate. No appreciable heating was observed during the $\upmu$s-PEF treatment.

First, we analyzed the effects after the application of pulses of increasing voltage (Fig. 3). The major effect was the displacement of the MT towards the anode (+). This anode migration effect was attributed to the overall negative charge of tubulin and MTs^11^ and has also been observed for lower voltages and unbound MTs^32,33^. To quantitatively analyze the pulse voltage dependence of this effect, we defined the *microtubule displacement index speed* (see “Development of measures for quantification of µs-PEF effects on microtubules”), with the results presented in Fig. 4e. As shown in the graph, the value of the MT displacement index speed was proportional to the applied pulse voltage. This finding is understandable, because the electric field is expected to act on MTs by a force, which is linearly proportional to the applied pulse voltage. However, substantial displacement of MTs starts to appear only from the threshold voltage of 210 V onwards. How can this threshold effect be explained? We propose that the most likely mechanism is that the electric field needs to overcome the antibody-mediated binding of MTs to the substrate. To detach any of the MTs, the electric field has to be higher than a certain threshold. The specific threshold value will likely depend on how many antibodies are holding the MTs at the surface (as a support of this concept, see atomic force microscopy analysis of ferritin-antiferritin antibody bond breaking force^34^), per MT length, which is statistically determined by the antibody surface density. We can see that substantial effects are seen from the applied voltage of approximately 210 V and up. From the applied voltage, we can also estimate the field strength, force, and estimated anti-body density; see “Forces underlying microtubule detachment” for the detailed quantitative discussion.

Another effect that we observed, as a consequence of the MT displacement, was the increasing overlap and concentration (accumulation) of MTs with increasing voltage (Fig. 3j). We propose the following mechanism behind this effect: when an electric field "sweeps" MTs to the region where the field is already not strong enough, further displacement of MTs is not possible anymore. The concentration of MTs in that region increases and they accumulate and overlap. Although MTs have a negative charge and are expected to repel each other, they are also surrounded by a counterion cloud that screens the mutual repulsion between MTs (calculated Debye length is approximately 0.7 nm for our experimental conditions, see supplementary material S1 and S8). A crowding agent, methyl cellulose, was used in our experiments to keep MTs at the imaging plane. If no methyl cellulose was present, MTs were not only detached from the surface by $\upmu$s-PEF but they were also removed from the image plane and disappeared from the field of view (see supplementary videos S6 and S7). Methyl cellulose, as other crowding agents, might also contribute to MT clumping and bundle formation in vitro^35,36^ owing to the depletion force that they exert. We note that the presence of methyl cellulose is a necessary, but not a sufficient condition to observe MTs accumulation and overlap. The electric field first has to not only detach, but also start to translocate the MTs to the side. Only in the course of translocation, MT accumulation started during the encounter of MTs and further accumulated over time. To quantify the MTs accumulation and overlap, we developed a measure termed *microtubule overlap rate*. Although an increasing overlap was observed with increasing voltage almost over the whole voltage range (Fig. 3j), a more pronounced increase occurred only from 210 V and above.

Furthermore, we analyzed the time dependence of the $\upmu$s-PEF effects on MTs during a sequence of pulses (Fig. 4). In this figure, t $=$ 0 s indicates the time at the start of pulsing. We selected to plot snapshots from the live imaging at the highest voltage settings (300 V) of the applied pulses (Fig. 4a–d) to qualitatively demonstrate the $\upmu$s-PEF effect on the MTs. The quantitative time dependence of MT motion during a pulsing is depicted in Fig. 4e (quantified by *microtubule displacement index speed*). We again observed the threshold effects: the appreciable motion of MTs occurred only from 210 V and upwards. Interestingly, close inspection of TIRF microscope imaging videos (see supplementary data, for example, 170 V video) revealed that a few individual MTs, which were probably only weakly (via fewer antibodies) attached to the substrate, start to migrate in the electric field even at lower voltages. Moreover, from 210 V upwards, the dynamics of MT movement is differentiated: the higher the voltage, the sharper the dynamics onset. And finally, above 255 V: the higher the voltage, the sooner all movable MTs were displaced. This can be understood based on the assumption that each MT has a particular value of binding energy to the substrate. The particular value of the MT-substrate binding energy determines the value of the threshold force (which in turn determines the value of the threshold electric field and voltage) that needs to be overcome to detach the particular MTs from the surface. That might explain the observed effects: for low voltages, only a few MTs were detached and displaced and as the voltage was increased, more MTs were detached and displaced. Hence, we suggest that both displacement and overlap measures are crucially dependent on the binding properties and hence the composition of the substrate.

**Figure 5:**
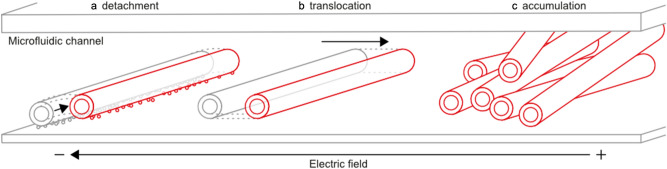
The scheme describing the on-chip effects of $\upmu$s-PEF on microtubules. **(a)** $\upmu$s-PEF detaches microtubules from the substrate—this effects requires high electric field strength, which would be unattainable in DC regime. **(b)** $\upmu$s-PEF translocates the microtubules by electrophoretic force towards the anode. **(c)** $\upmu$s-PEF accumulates microtubules at the area where the electric field strengths drops below a threshold level capable of detaching and translocating microtubules.

In summary, in the current work, a $\upmu$s-PEF was employed to: (i) detach MTs from the substrates, which requires a high field strength, (ii) move MTs (also possible with a weaker field), and (iii) accumulate MTs due to the particular field distribution. See Fig. 5 for a schematic representation of these effects.

### Forces underlying microtubule detachment

Here we express forces underlying the electrical detachment of MTs from the surface and what can be learned from them. The force that pulls MTs is the electric field force $F_E = Q \cdot E$, where $Q_{MT}$ is the charge of the MT and *E* is the electric field strength. There are typically $N=$ 13 protofilaments in MT and the tubulin heterodimer of the $L_{TUB} =$ 8 nm length has effectively around $Q_{TUB}$ = − 23 *e* (elementary charges)^32^. Therefore, the $Q_{MT} = L_{MT} \cdot \rho _{MT}$, where $L_{MT}$ is the MT length and $\rho _{MT} = N \cdot Q_{TUB} \cdot L_{TUB}^{-1}$ is the linear density of charge on MT. Assuming that the force that holds the MT at the surface due to antibody binding is $F_{HOLD} = D_{AB} \cdot L_{MT} \cdot F_{AB}$, where $D_{AB}$ is the linear density of the antibody molecules connecting the MT to a surface (per MT length) and $F_{AB}$ is the holding force per antibody. Comparing $F_{HOLD} = F_E$, one can express the underlying quantities, provided the values of other quantities are known or can be reasonably assumed. For example, one can express $D_{AB} = E \cdot \rho _{MT} \cdot F_{AB}^{-1}$. Assuming that the force needed to detach a single antibody from a microtubule $F_{AB}$ is 50 pN^34^, then a MT detached for example at 210 V (17.5 kV/cm, see Fig. 1e) corresponds to approximately 210 antibodies which bind MT to the substrate per 1 $\upmu$m of MT length (considering the MT footprint of 25 nm (MT width) multiplied by MT length). This seems to be a reasonable estimate, since the comparable antibody density (6400 antibodies/$\upmu$m$^2$, which corresponds to 160 antibodies / $\upmu$m of MT length) used in functionalization assays for comparable concentration and type of antibodies (1 mg/mL in this work vs. 0.5 mg/mL in^37^, both IgG1) has been also reported elsewhere^37^. See S8 for the details of the calculation.

### Advances, future challenges and opportunities

PEF technology is becoming widespread in industry and research, in which it is commonly used to target membrane structures and leverage their permeabilization for various purposes. Comparatively, the effects of $\upmu$s-PEF on protein structures have been much less explored, particularly on a single molecule level. Therefore, the main advances to the state-of-the-art of our current work include (i) the demonstration of the $\upmu$s-PEF technology embedded in a TIRF microscope and (ii) the threshold-based, simultaneous actuation of MTs mediated by $\upmu$s-PEF, which is capable of overcoming the molecular non-covalent binding forces holding the MTs at the substrate. Furthermore, our chip design enables the exploration of electric field effects on MTs minimizing (here unwanted) complex electrochemical phenomena that occur in the electrode/electrolyte interface. This is achieved by delivering a strong electric field throughout the volume of the microfluidic channel. In contrast to our recent work^20^, the current platform is scientifically and methodically distinct: (i) a whole new and very different microscope stage holder and insert were designed for the current microscope method (TIRF), which has never been combined with PEF chips, (ii) the $\upmu$s-PEF chip assembly was developed to be compatible with an *in vitro* MT system instead of cells, (iii) we used spacers (double coated tape) to define the height of the microfluidic channel, and (iv) no electroplating was carried out on the thin-film deposited metal to achieve a higher control of the metal pattern dimensions and to keep the electrode surface smooth.

We selected TIRF microscopy in a broader context of our research aiming to explore electromagnetic field effects on the scale of biomolecules. Particularly, because a TIRF microscope is capable of single molecule imaging^38^, in future work our technology will be harnessed for simultaneous imaging and $\upmu$s-PEF-modulation of the function of MT-associated proteins (MAPs) such as motor proteins.

Using our on-microscope chip platform, we demonstrated the possibility that protein structures, such as MTs, could be affected by an $\upmu$s-PEF. This adds to the evidence accumulated from both computational simulations^39,40^ and experiments^41,42^ that a particularly intense electric field (typically > MV/m) can influence protein structures, including tubulin^11,43^. These strong indications - that intense PEF could affect proteins—paint a more complex picture of the PEF effects on cells, since mostly membranes have been the focus as a target of a PEF^14^.

In future work, our findings might also be leveraged in bio-nanotechnological applications that require MT manipulation. So far, MT manipulation has been achieved by dielectrophoretic effects using an alternating electric field (frequencies up to 10 MHz)^44–46^ or long electric pulses (tens of seconds to minutes) using a static electric field (direct current)^32,33,47^, and often in combination with kinesin-coated surfaces that power the MTs’ motion^44,47–49^. There are several features in our current work that were not demonstrated in these previous works and techniques. The major one is that we were able to achieve highest electric field strength reported so far in MT electro-manipulation. This is important because the high field strength is needed for the detachment of MTs bound to a substrate via antibodies.

A dielectrophoretic approach, which employs AC fields, is unlikely to achieve sufficiently high forces to detach MTs throughout the volume of the electrode gap. To achieve a high force and the same effect by dielectrophoresis requires: (1) a high field gradient, which is only local at the edges of the electrodes, (2) often a field strength with a magnitude that is 3–4 orders of higher than the direct electric field effect on charges^46^.

A high field strength could not be achieved using a static electric field (longer duration than seconds) for practical reasons: for example, in our conditions, the application of even several volts generated bubbles by electrolysis within a few seconds. There would also be significant heating for higher voltages at the seconds and longer timescales.

Therefore, strong-field-mediated effects for MT manipulation, without heating, can be achieved by employing short, intense electric pulses, as demonstrated here. For this approach, it would be useful to control the effective electric charge of the MTs: either by buffer composition^50^, by protein sequence^51^, or by certain post-translational modifications^52^, which add basic or acidic residues, such as glutamic acid^53^, by the removal of the negatively charged C-terminal tail by subtilisin treatment^32^, or by binding other charged molecules, such as DNA^48^.

What are the potential biological and biophysical implications of our findings of external electric field acting on microtubule network? It is clear that electrostatic interactions are crucial in the energy balance determining microtubule stability^54^ and also for interactions with other proteins^55^. The potential contribution of electrostatics to microtubule alignment and organization *in vivo* is a much more complex question. Such a role would be indirect through the interaction with motor proteins and other cross-linking proteins that enable the MT organization, for example in mitosis. An external electric field could potentially disrupt the interaction between microtubule and kinesin^40^, which could hypothetically lead to direct disruption of organized MT systems.

At the cellular level and in medical applications, the standardized protocols of PEF based-treatments use 100 $\upmu$s as pulse duration^56,57^. Because 100 $\upmu$s is well above the charging time of the cell membrane for physiological extracellular conductivity values^58^, it leads to the electroporation of the cell membrane as a primary phenomenon. In contrast, shorter pulses at the nanosecond scale affect several intracellular components simultaneously with the electroporation of cell membrane and lead for example to the disruption of individual cytoskeleton filaments^21^. In the current paper, we investigated the effect of the intermediate length of electric pulses, where we found that the integrity of individual cytoskeleton filaments could be preserved. Thus, we demonstrated that in this range of PEF duration, the entire dense MTs network could be translocated collectively towards the anode, effectively simulating PEF effects on various noncovalent interactions of individual filament with other subcellular components. This phenomenon is of great importance in biophysical processes which involve cell mechanics, migration, and polarization.

## Conclusion

We developed a chip platform that enables the delivery of an intense pulsed electric field to MT systems while enabling live imaging of MTs with a TIRF microscope. Using this new chip platform, we showed that the binding of MTs to the substrate can be disrupted, resulting in the release and actuation of the MTs. These proof-of-concept results suggest an intense $\upmu$s-PEF can be used to manipulate non-covalent bonding of charged biomolecular systems, which was demonstrated by investigating the interactions between MTs and their binding partner. We showed that a high-strength $\upmu$s-PEF is required, at least in the order of MV/m, to affect those non-covalent interactions. These results contribute to the knowledge of PEF biological effects in the poorly explored region of $\upmu$s-scale PEF. We believe that our in vitro results will stir interest to explore similar $\upmu$s-PEF parameters in vivo to influence MT interactions. Overall, our results bring new technology for studying field-matter interaction, data on $\upmu$s-PEF effects on MT systems and expand the toolbox of bioelectronic approaches for nanotechnology and biomedicine.

## Materials and methods

### Chip fabrication

The fabrication of a chip is based on a lift-off process with maskless direct optical lithography (Quantum Design Inc., MicroWriter ML^®^2). We used a quartz glass substrate purchased from Technical Glass Products, Inc. with dimensions of 25.4 × 25.4 × 1 mm$^3$. The slide was thoroughly cleaned with acetone, ethanol, and pure water (Millipore^®^, Direct-Q^®^ 3 UV with Pump | ZRQSVP030, $\sigma$ = 0.05 $\upmu$S/cm @22 °C) and ozone cleaner was used for 10 min just before spin coating. The cleaned slide was first spin-coated with LOR 5A lift-off resist (MicroChem Corp., pre-bake and soft-bake 190 °C for 5 min) and followed by a layer of S1818^TM^ G2 positive photoresist (Microposit^TM^, 120 °C for 2 min). The pattern was transferred using a maskless direct optical lithograph with an exposure dose of 160 mJ/cm$^2$ @ 1 $\upmu$m 405 nm laser. After exposure, the post-bake procedure consisted of heating to 120 °C for 1 min and cooling down in the air for 2 min. Then, it was developed in MF-319 developer (Microposit^TM^ MF^TM^-319 developer) for approximately 1 min with slow agitation. The chip metallization consisted of a 20 nm layer of evaporated chromium as an adhesion layer with 300 nm gold on the top. The evaporation was followed by a lift-off process by immersing into mr-Rem 700 remover solution (Micro resist technology GmbH, 55 °C) for stripping of the resist. Afterwards, the slide was thoroughly rinsed with pure water and ethanol and then dried with nitrogen (Fig. 2 a)).

### Microtubule sample preparation and buffer for experiments

Tubulin was isolated from pig brains obtained from local slaughterhouse and labeled as described previously^59^. For the preparation of fluorescently labeled biotinylated MTs for a TIRF microscope assay, unlabeled pig brain tubulin was mixed with HiLyte640™dye labeled tubulin (Cytoskeleton Inc., TL670M-A) and biotinylated tubulin (Cytoskeleton Inc., T333P) to 4 mg/mL at a 68:20:12 mass ratio. 2.5 $\upmu$L of the tubulin mixture was diluted in 5 $\upmu$L BRB80 buffer (80 mM PIPES, 1mM EGTA, 1mM MgCl2, pH 6.8) supplemented with GMPCPP (2.7 mM final concentration) and incubated for 5 min on ice. GMPCPP was used to prevent depolymerization of the MTs. The MTs were polymerized for 30 min at 37 °C and then centrifuged at 14,000 ×  g in a tabletop centrifuge for 5 min at room temperature (22 °C) to remove any unpolymerized tubulin. The MT pellet was subsequently resuspended in 50 $\upmu$L BRB80 and used within one week. The buffer used in the experiments was BRB6 supplemented with 20 mM D-glucose (Sigma-Aldrich, G7528), 22.4 mg/mL glucose oxidase (Sigma-Aldrich, G2133), 20 mg/mL catalase (Sigma-Aldrich, C9322), and 0.22% (w/v) methyl cellulose (Sigma-Aldrich, M0512) - the crowding agent.

### Imaging setup and settings

Imaging was performed at room temperature (22 °C) using an inverted microscope (Nikon, Ti-E Eclipse) with a 60 × 1.49 N.A. oil immersion objective (Nikon, Plan Apo) and an EMCCD camera (Andor Technology, iXon Ultra 888). Samples were excited using LU-N4/N4S laser unit (Nikon). Illumination and image acquisition was controlled by NIS Elements Advanced Research software (Nikon). MTs were illuminated using a 647 nm laser. Time-lapse images were acquired at a frame rate of 2.47 FPS with an exposure time of 100 ms.

### Fluidics assembly and channel functionalization

Microflow chambers for the TIRF microscope MT assay were prepared by attaching a cleaned and hydrophobisized 18 × 18 mm$^2$ coverslip (the treatment procedure is described previously^60^) on the top of the chip using 50 $\upmu$m thick double coated tape strips (Nitto, Double-coated Tape 5005P), which also determined the channel height. This thickness allowed for a homogeneous electric field throughout the channel, as indicated in Fig. 1e,d. The channel was treated with anti-$\beta$-tubulin (Sigma-Aldrich, T7816, 1 mg/mL in PBS) antibody solution for 5 min, followed by one-hour incubation with 1% Pluronic F127 (Sigma-Aldrich, P2443). MTs were flushed in the channel before the experiment and incubated for 1 min to let MTs to attach to the substrate via antibodies. Before the experiments, the channels were perfused with the BRB6 buffer, see the section earlier. For the pulsed electric field setup, see the Results section and Fig. 2.

## Supplementary Information

Supplementary Information.

Supplementary Figure 1.

Supplementary Figure 2.

Supplementary Figure 3.

Supplementary Figure 4.

Supplementary Video S1.

Supplementary Video S2.

Supplementary Tables.

## Data Availability

The data that support the findings of this study are available within the article and its supplementary material.
