# Supplementary material for: Lab-on-chip microscope platform for electro-manipulation of a dense microtubules network
Source: Sci Rep. 2022 Feb 14;12:2462. doi: 10.1038/s41598-022-06255-y (PMC8844285; doi:10.1038/s41598-022-06255-y)
Supplement: Supplementary file 1 — Supplementary Information. [file 41598_2022_6255_MOESM1_ESM.pdf]

# Supplementary information S1 for: Lab-on-chip microscope platform for electro-manipulation of a dense microtubules network

Daniel Havelka[a,e], Ilia Zhernov[b], Michal Teplan[c], Zdeněk Lánský[b], Djamel Eddine Chafai[d,a], Michal Cifra[a,e]

[a]*Institute of Photonics and Electronics of the Czech Academy of Sciences, Prague, Czechia*
[b]*Institute of Biotechnology of the Czech Academy of Sciences, Prague, Czechia*
[c]*Institute of Measurement Science of the Slovak Academy of Sciences, Bratislava, Slovakia*
[d]*Institute of Physiology of the Czech Academy of Sciences, Prague, Czechia*
[e]*corresponding authors: cifra@ufe.cz, havelka@ufe.cz*

---

---

## 1. List of supplementary materials with legend

- S1: this document
- S2 – S7: microscopy videos, see Table S1.1 lower for details
- S8: .xls file with analytical calculation of Debye length and electric field effect on detachment

Table S1.1: List of raw data and supplementary videos. BRB6 is the full buffer as described in the main text. BRB12 indicates PIPES concentration of 12 mM instead of 6 mM. mc = 0.22 % w/v methyl cellulose

| | | | electric pulse settings | | | | |
|---|---|---|---|---|---|---|---|
| video label | source file | experiment. day | voltage [V] set / measured | number of pulses | firing frequency [Hz] | width [μs] | buffer |
| - | 1 | 20200909 | 50/45 | 100 | 10 | 5 | BRB6,mc |
| - | 2 | 20200909 | 100/85 | 100 | 10 | 5 | BRB6,mc |
| - | 3 | 20200909 | 150/125 | 100 | 10 | 5 | BRB6,mc |
| S2 | 4 | 20200909 | 200/170 | 100 | 10 | 5 | BRB6,mc |
| S3 | 5 | 20200909 | 250/210 | 100 | 10 | 5 | BRB6,mc |
| S4 | 6 | 20200909 | 300/255 | 100 | 10 | 5 | BRB6,mc |
| S5 | 7 | 20200909 | 350/300 | 100 | 10 | 5 | BRB6,mc |
| S6 | 9 | 20200731 | 400 | 100 | 10 | 5 | BRB12 |
| S7 | 11 | 20200819 | 150 | 100 | 10 | 5 | BRB6 |

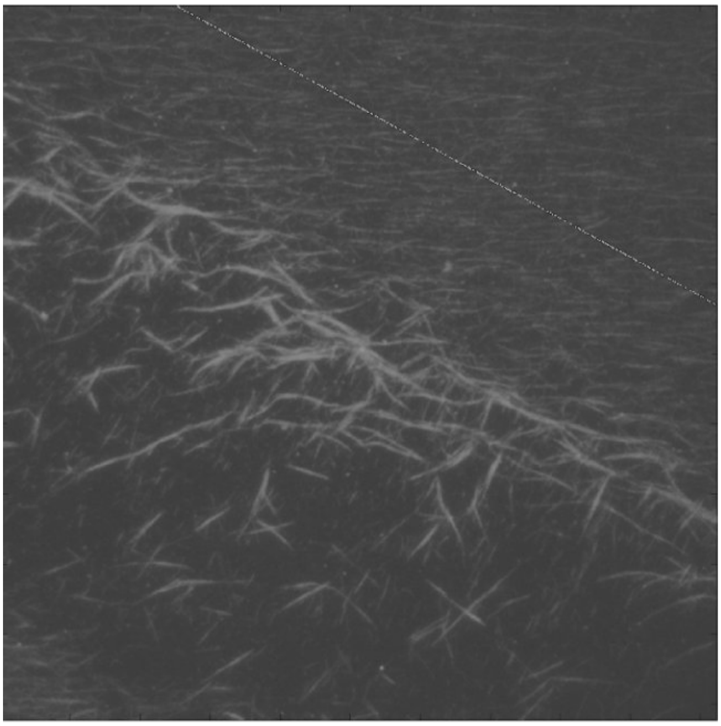

Figure S1.1: Background = area unaffected by μs-PEF situated to the right from the border line. ROI = area to the left and bellow.

## 2. Image analysis and measure development details

## 3. Raw data

The raw data (videos in .nd2 Nikon format) are available on: `https://doi.org/10.5281/zenodo.4543544`.

All videos are from TIRF imaging as described in the main text. For the analysis in the main text, we used video files 1-7 (experimental day 20200909 - formatted as YYYYMMDD). The further videos are: video file 9 from experimental day 20200731 and video file 11 from experimental day 20200819.

## 4. Supplementary videos

Selected videos are also provided as supplementary videos in .mp4 format, see Table 1.

Data from videos S6 and S7 were not used for any of the quantitative analysis in the manuscript, they are just to demonstrate the detachment of microtubules from the substrate under the effect of PEF, when there was no methyl cellulose in the sample.

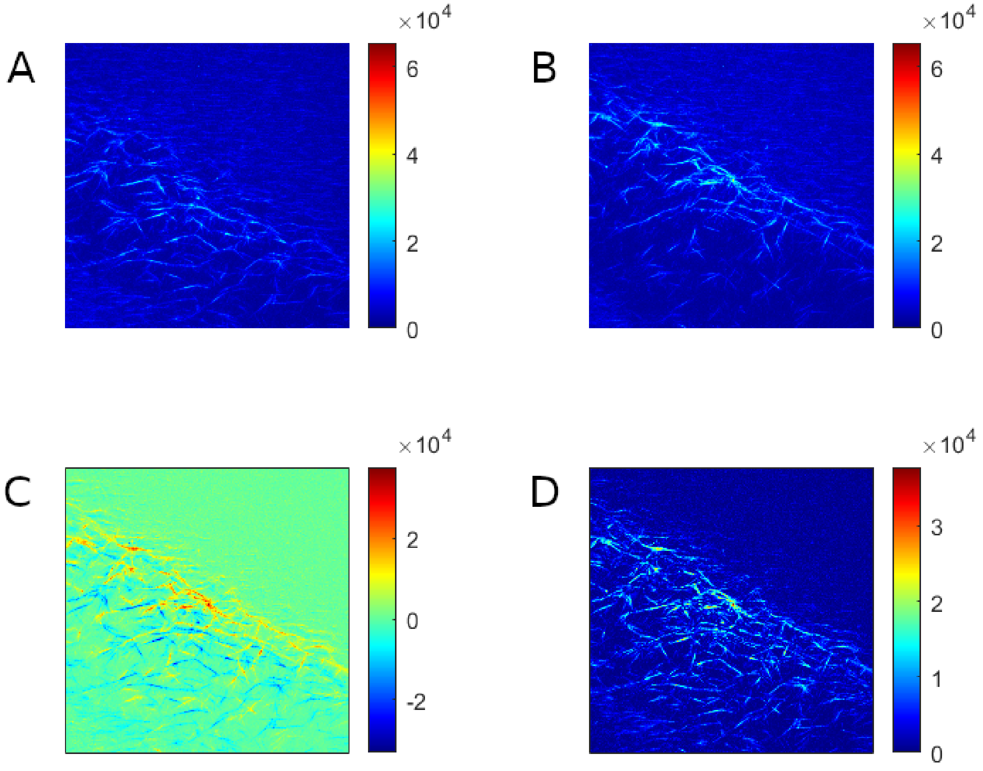


Figure S1.2: Illustration of the mechanism behind the measure *microtubule displacement index*: TIRF microscope intensities prior (A) and after (B) µs-PEF, a difference of intensity matrices (C) with appearance of MTs at the red and yellow spots and disappearance of MTs at the blue spots, and a difference of absolute values of intensity matrices (D) that was used in the formula in order to cover the both types of changes.

## 5. Debye length calculation

The Debye length, a measure of the distance at which the3 electric potential from a source will decrease to 1/e due to screening of mobile charges, is given as [1]

$$\lambda_D = \sqrt{\frac{\epsilon\epsilon_0 \mathrm{k} T}{\sum_i \rho_i e^2 z_i}} \tag{S1.1}$$

where $\epsilon$ is the relative permittivity of the medium (here we consider water-like, i.e. $\epsilon$=78), $\epsilon_0$ is the permittivity of the vacuum, k is Boltzmann constant, T is temperature (considering 298 K), $\rho_i$ is the number density of the charge (ion), $z_i$ is the ion valency e is elementary charge. The Debye length can be also expressed as [2]

$$\lambda_D = \sqrt{\frac{\epsilon\epsilon_0 \mathrm{k} T}{2 e^2 N_A I}} \tag{S1.2}$$

where $N_A$ is Avogadro's constant, and I is ionic strength [3][p.259]

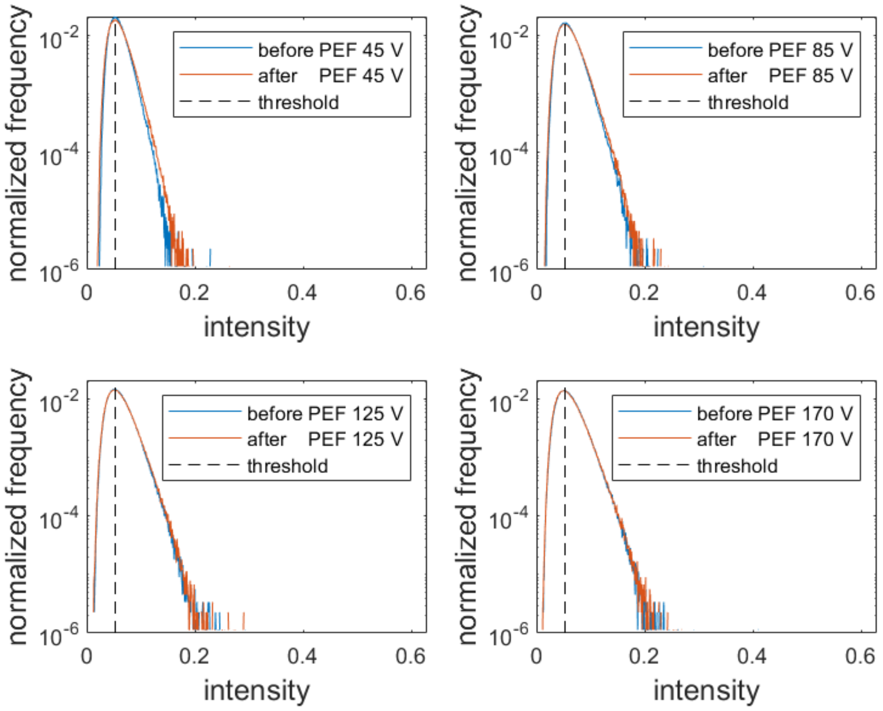


Figure S1.3: Clarification of *microtubule overlap rate*: Histograms of TIRF intensities prior and after µs-PEF for 4 lower voltages.

$$I = \frac{1}{2} \sum_i c_i z_i \tag{S1.3}$$

where $c_i$ is concentration of the ion in mol/m$^3$.

Expression in Eq. S1.2 is useful because I can be approximately obtained from the measured electrical conductivity using a conversion coefficient. This coefficient is between 0.13 – 0.17 dS/m for 1 M (moles/L) of ionic strength [4]. Assuming the conversion coefficient of 0.16, for the conductivity of our experimental media $\sigma$ = 0.1188 S/m, the corresponding ionic strength is 0.19 M or 190 mol/m$^3$. Under these conditions, the calculated Debye length is 0.7 nm, see calculations in S8.

## 6. Comment on electrolysis in experiments

One of the major limitations of the current approach is the formation of bubbles caused by electrolysis of the buffer, which ultimately limited the number of electric pulses that could be delivered and the length of time that the MTs could be exposed. This limitation could be solved in future work by using a fully throughflow microfluidic channel connected to external reservoirs with degassed buffers. In such a way, the buffer could be continuously replaced, removing the gas

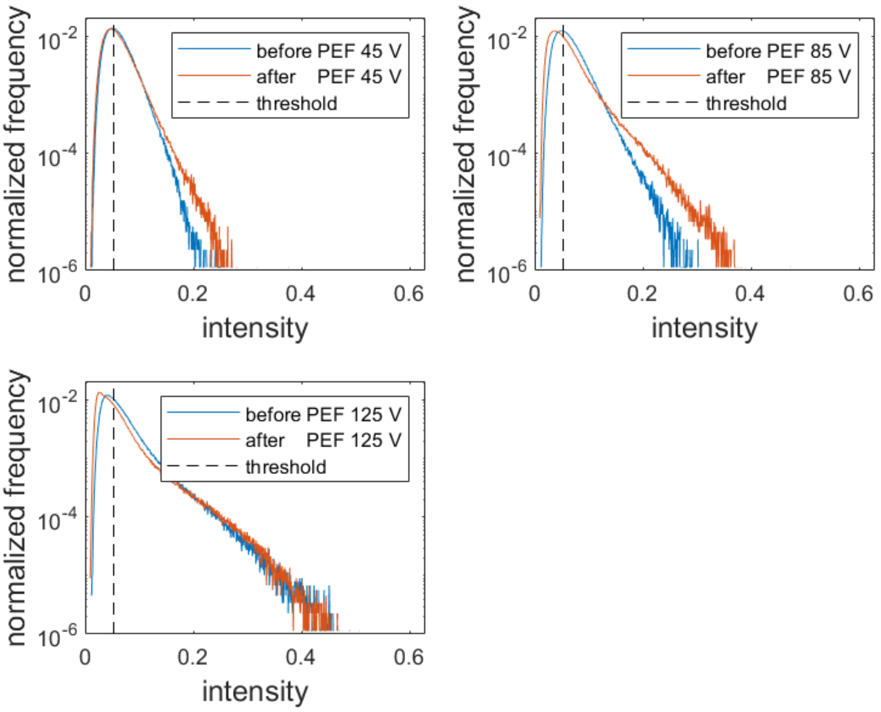


Figure S1.4: Clarification of *microtubule overlap rate*: Histograms of TIRF intensities prior and after µs-PEF for 3 higher voltages. Increased right tail of distribution after µs-PEF in the case of 210 and 255 V is caused by intensity elevation in a limited number of points in ROI area due to overlap of MTs. On the other hand, decreased left tail of distribution after µs-PEF in the case of 255 and 300 V is caused by relative emptying of ROI space. For this analysis, MTs are defined as objects consisting from the pixels with intensities above the depicted threshold.

being formed. However, another solution that could be used to prevent the formation of bubbles is to avoid the charge transfer between the electrodes and the buffer. This could be achieved by insulating the electrodes and delivering the electric field to the channel via capacitive coupling, which would be most effective for pulses with nanosecond-scale duration or shorter.

## References

[1] Alexander M. Smith, Alpha A. Lee, and Susan Perkin. The Electrostatic Screening Length in Concentrated Electrolytes Increases with Concentration. *The Journal of Physical Chemistry Letters*, 7(12):2157–2163, June 2016.

[2] Zhi Zheng, Hongyuan Zhang, Tianyou Zhai, and Fan Xia. Overcome Debye Length Limitations for Biomolecule Sensing Based on Field Effective Transistors †. *Chinese Journal of Chemistry*, 39(4):999–1008, April 2021.

[3] John O'M Bockris and Amulya K.N. Reddy. *Modern electrochemistry - ionics*, volume 1. 1998.

[4] M. Simón and I. Garcia. Physico-chemical properties of the soil-saturation extracts: estimation from electrical conductivity. *Geoderma*, 90(1-2):99–109, June 1999.
