# Supplementary figures and images for: Lab-on-chip microscope platform for electro-manipulation of a dense microtubules network
Source: Sci Rep. 2022 Feb 14;12:2462. doi: 10.1038/s41598-022-06255-y (PMC8844285; doi:10.1038/s41598-022-06255-y)

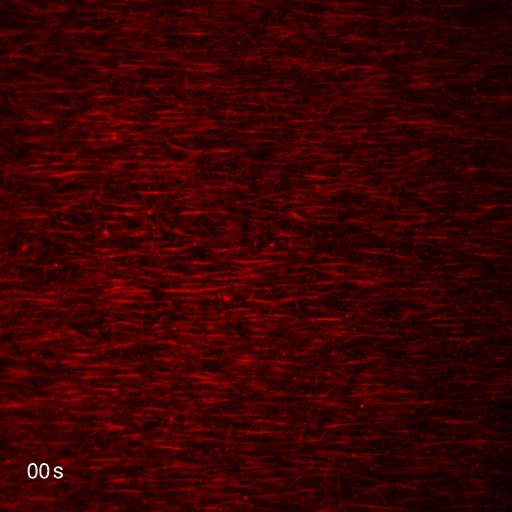

Supplement: Supplementary file 2 — Supplementary Figure 1. [file 41598_2022_6255_MOESM2_ESM.gif]

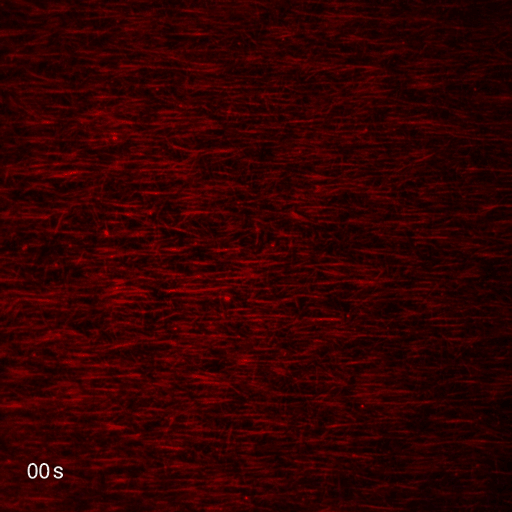

Supplement: Supplementary file 3 — Supplementary Figure 2. [file 41598_2022_6255_MOESM3_ESM.gif]

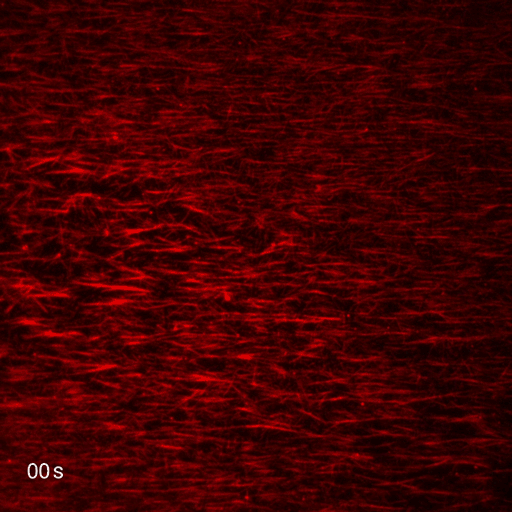

Supplement: Supplementary file 4 — Supplementary Figure 3. [file 41598_2022_6255_MOESM4_ESM.gif]

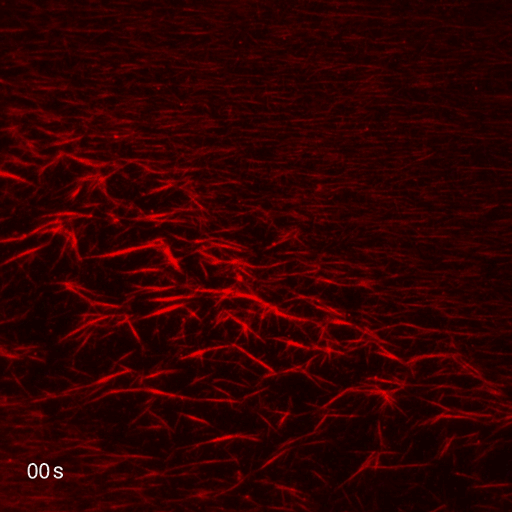

Supplement: Supplementary file 5 — Supplementary Figure 4. [file 41598_2022_6255_MOESM5_ESM.gif]
